# *Circ-NOLC1* promotes epithelial ovarian cancer tumorigenesis and progression by binding ESRP1 and modulating CDK1 and RhoA expression

**DOI:** 10.1038/s41420-020-00381-0

**Published:** 2021-01-22

**Authors:** Shuo Chen, Wu Wu, Qian-hui Li, Bu-min Xie, Fan Shen, Yu-ping Du, Zhi-hong Zong, Li-li Wang, Xiao-qing Wei, Yang Zhao

**Affiliations:** 1 Department of Gynecologic Oncology Research Office, The Third Affiliated Hospital of Guangzhou Medical University, Guangzhou, 510150 China; 2 Department of Obstetrics and Gynecology, Center for Reproductive Medicine/Department of Fetal Medicine and Prenatal Diagnosis, Key Laboratory for Major Obstetric Diseases of Guangdong Province, The Third Affiliated Hospital of Guangzhou Medical University, Guangzhou, 510150 China; 3 Department of Gynecology, The First Affiliated Hospital of China Medical University, Shenyang, 110001 China; 4 Oral and Biomedical Sciences, School of Dentistry, Cardiff University, CF14 4XY Cardiff, UK

**Keywords:** Ovarian cancer, Molecular biology

## Abstract

Circular RNAs (circRNAs) play important roles in cancer tumorigenesis and progression, representing prognostic biomarkers and therapeutic targets. In this case, we demonstrated the role of *circ-NOLC1* in epithelial ovarian cancer (EOC). Our results have shown that *Circ-NOLC1* expression was higher in EOC tissues than in normal tissues, and was positively associated with FIGO stage, differentiation. Among ovarian cancer cell lines, *circ-NOLC1* expression was the highest in A2780, and lowest in CAOV3. Overexpression of *circ-NOLC1* in CAOV3 cells increased cell proliferation, migration, and invasion ability, whereas silencing of *circ-NOLC1* in A2780 cells had the opposite effect: however, neither *circ-NOLC1* downregulation nor overexpression influenced *NOLC1* mRNA expression. In nude mice with subcutaneous tumors, *circ-NOLC1* downregulation decreased tumor growth. Bioinformatic analysis and RNA-binding protein immunoprecipitation showed that *circ-NOLC1* could bind to ESRP1. In addition, the overexpression of *circ-NOLC1* significantly increased ESRP1, RhoA, and CDK1 protein and mRNA expression level; *circ-NOLC1* downregulation had the opposite effects. The tumor-promoting effect of *circ-NOLC1* was inhibited by knockdown of ESRP1, CDK1, or RhoA expression in *circ-NOLC1*-overexpressing cells, which might act by modulating RhoA and CDK1 expression. In conclusion, our study demonstrated that *Circ-NOLC1* might promote EOC tumorigenesis and development by binding ESRP1 and modulating CDK1 and RhoA expression.

## Introduction

Ovarian cancer is a malignant tumor that seriously threatens women’s health^1–3^. Asymptomatic clinical appearance in the disease’s progression, resulting in widespread disease in the pelvic and abdominal cavity. Therefore, even though surgery, chemotherapy, and biological therapy are used widely to treat ovarian cancer, the 5-year survival rate remains at only 35–38%^4,5^. Thus, research into the mechanism of ovarian cancer development is important for its early detection, diagnosis, and treatment.

The occurrence and development of ovarian cancer is a complex process, comprising multi-step, multi-gene, and multi-factor regulations, especially the regulation of transcription factor regulatory networks, signaling pathways, and regulatory loops. Recent studies have shown that non-coding RNAs also play an important role in the process of ovarian cancer proliferation, apoptosis, invasion, and metastasis^6^.

Non-coding RNAs are RNA molecules in the transcriptome that are not translated into proteins, including small nuclear RNAs, small nucleolar RNAs, microRNAs with relatively small molecular weight (miRNAs), piwi-interacting RNAs, long non-coding RNAs, and circular RNAs (circRNAs) with relatively large molecular weights. CircRNAs are endogenous RNAs that are widely distributed in eukaryotic cells and have a stable cyclic structure, playing a vital role in the regulation of gene expression. Their unique covalently closed loop structure means that they lack a 5′-end cap structure and 3′ terminal polyadenylation (polyA) tail, which is characterized by tolerance to exonucleases and makes them more stable than homologous linear RNA^7–9^. CircRNAs play vital roles in almost all kinds of cancer and have the potential to be used as novel biomarkers and therapeutic targets for cancer diagnosis and treatment through their functions as microRNA (miRNA) molecular “sponges”^10–12^, protein translators^13^, and gene transcription regulators^14^. For example, *circTCF25* overexpression promoted cell proliferation and migration by downregulating miR-103a-3p/-107 expression and increasing *CDK6* (cell-dependent kinase 6) expression in vitro or in vivo^11^. Recent studies also revealed that circRNAs could function as RNA-binding protein sponges^15^. For example, *circ-Foxo3* can inhibit CDK2 (cell-dependent kinase 2) and induce cell cycle arrest through a circ-Foxo3-p21-CDK2 ternary complex^16^. However, there are relatively few studies on circRNAs in ovarian cancer.

We detected 12,723 expressed circRNAs in ovarian cancer and normal ovarian tissue through circRNA gene chip, and further screened circRNAs with differential expression up to fivefold, which identified *circ-NOLC1*. Nucleolar and coiled-body phosphoprotein 1 (NOLC1, also known as Nopp140) is a protein with transcription-like activity, which involves in nuclear assembly^17^ and precursor rRNA cleavage^18^, as a novel nucleolar GTPase/ATPase, it was reported to have a role in the process of the tumorigenesis and progression such as cell cycle, cell proliferation^19^, cell migration, and invasion. Similar to linear RNA, circRNA is also derived from the transcription of parental genes, and its uniqueness lies in the alternative splicing of the non-classical form of RNA precursors. The most common one is exon reverse splicing, that is, circRNA molecules is generated by back-splicing, where downstream exons are spliced to upstream exons in reverse order, leading to a circular transcript^20^. According to gene chip, the precursor RNA of NOLC1 can also form exonic circRNA circ-NOLC1 at exon 1–4 through back-splicing. However, the circular structure of *circ-NOLC1*, its relationship with NOLC1, and the most important, its role in ovarian cancer tumorigenesis and progression remains unclear.

Therefore, in the present study, we investigated the role of *circ-NOLC1* in ovarian cancer and attempted to determine how *circ-NOLC1* affects the development of ovarian cancer through a series of in vivo and in vitro experiments.

## Results

### The relationship between the circ-NOLC1 expression level and the clinicopathological characteristics of ovarian cancer

**Fig. 1:**
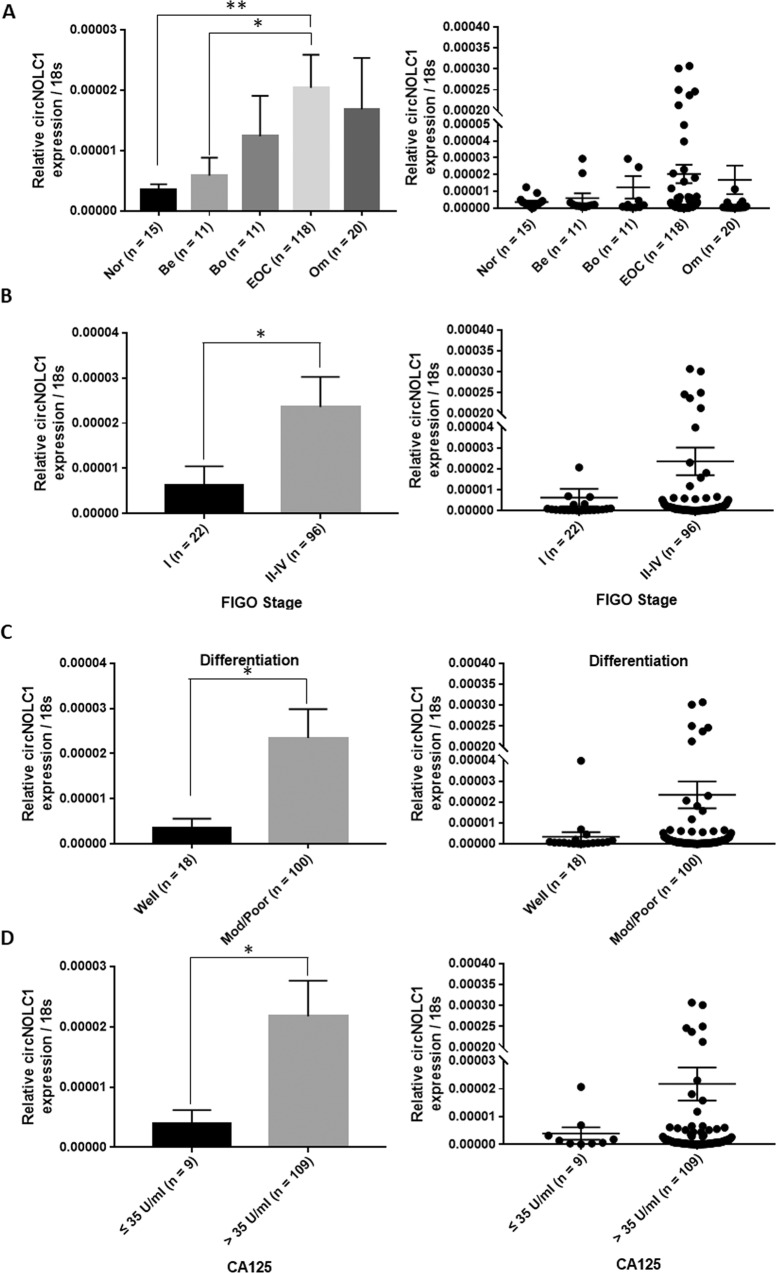
The relationship between circ-NOLC1 expression and the clinicopathological characteristics of ovarian cancer. **A** *Circ-NOLC1* expression in normal ovaries (*n* = 15), benign ovarian tumors (*n* = 11), borderline tumors (*n* = 11) and ovarian carcinomas (*n* = 118) was analyzed by qRT-PCR. **B** FIGO stages: The expression of circ-NOLC1 in stage II–IV (*n* = 96) was higher than that in stage I (*n* = 22). **C** Pathology classification: *circ-NOLC1* expression in the moderate (Mod) and Poor differentiation (*n* = 100) was higher than in the well differentiation (*n* = 18). **D** CA125: *circ-NOLC1* expression in the CA125 positive (*n* = 109) was higher than in the CA125 negative (*n* = 9) patients. Data are expressed as the mean ± SD. Correlation between circ-NOLC1 expression and clinicopathological characteristics were examined using the *χ*^2^ test. *p < 0.05.

*Circ-NOLC1* expression in normal ovarian tissues, benign tissues, borderline tumor tissues, and ovarian carcinoma tissues was assessed using quantitative real-time reverse transcription PCR (qRT-PCR). The results showed that *circ-NOLC1* expression was significantly higher in borderline and ovarian cancers than in normal and benign ovarian tissues (Fig. 1A, *p* < 0.05, Table 1). *Circ-NOLC1* expression in stage II–IV disease was higher than in stage I (Fig. 1B, *p* < 0.05). In addition, the expression of *circ-NOLC1* in the moderately and poor pathological differentiation was higher than that in the well differentiation (Fig. 1C, *p < 0.05). Besides, the expression of *circ-NOLC1* was also higher in CA125 positive (>35 U/ml) ovarian cancer patients (Fig. 1D, *p < 0.05, Table 2). These results suggested that *circ-NOLC1* involves in the tumorigenesis and progression of ovarian cancer, and may have potential to be treated as diagnostic biomarker.

**Table 1:** circ-NOLC1 expression in different ovarian tissues.

Groups	*N*	TDRG1 expression/18 s	*P* value
Normal ovary	15	3.57013E-06 ± 3.29678E-06	***0.0015***
Benign ovarian tumor	11	5.86156E-06 ± 9.77477E-06	***0.0108***
Borderline ovarian tumor	11	1.23827E-05 ± 2.21106E-05	
Ovarian carcinoma	118	2.03776E-05 ± 5.94288E-05	
Omentum tumor	20	1.68522E-05 ± 3.81715E-05	

Bold and italics means *P* < 0.05.

**Table 2:** Correlation of circ-NOLC1 expression with different clinicopathological features of ovarian carcinoma.

Clinicopathological features	*N*	circ-NOLC1 expression/18 s	*P* value
*The pathology types*			0.2795
Serous carcinoma	86	2.20741E-05 ± 6.38584E-05	
The other pathology types	32	1.58183E-05 ± 4.60309E-05	
*Age*			0.2531
≤52	62	2.38226E-05 ± 6.41964E-05	
>52	56	1.65634E-05 ± 5.39825E-05	
*CA125*			***0.0029***
≤35 U/ml	9	3.97132E-06 ± 6.6421E-06	
>35 U/ml	109	2.17322E-05 ± 6.16323E-05	
*FIGO stages*			***0.0144***
I	22	6.29372E-06 ± 1.94561E-05	
II–IV	96	2.36051E-05 ± 6.48809E-05	
*Pathology classification*			***0.0018***
Well	18	3.39141E-06 ± 9.26029E-06	
Mod + poor	100	2.34351E-05 ± 6.40101E-05	

Bold and italics means *P* < 0.05.

### circ-NOLC1 functions independently from the NOLC1 gene

**Fig. 2:**
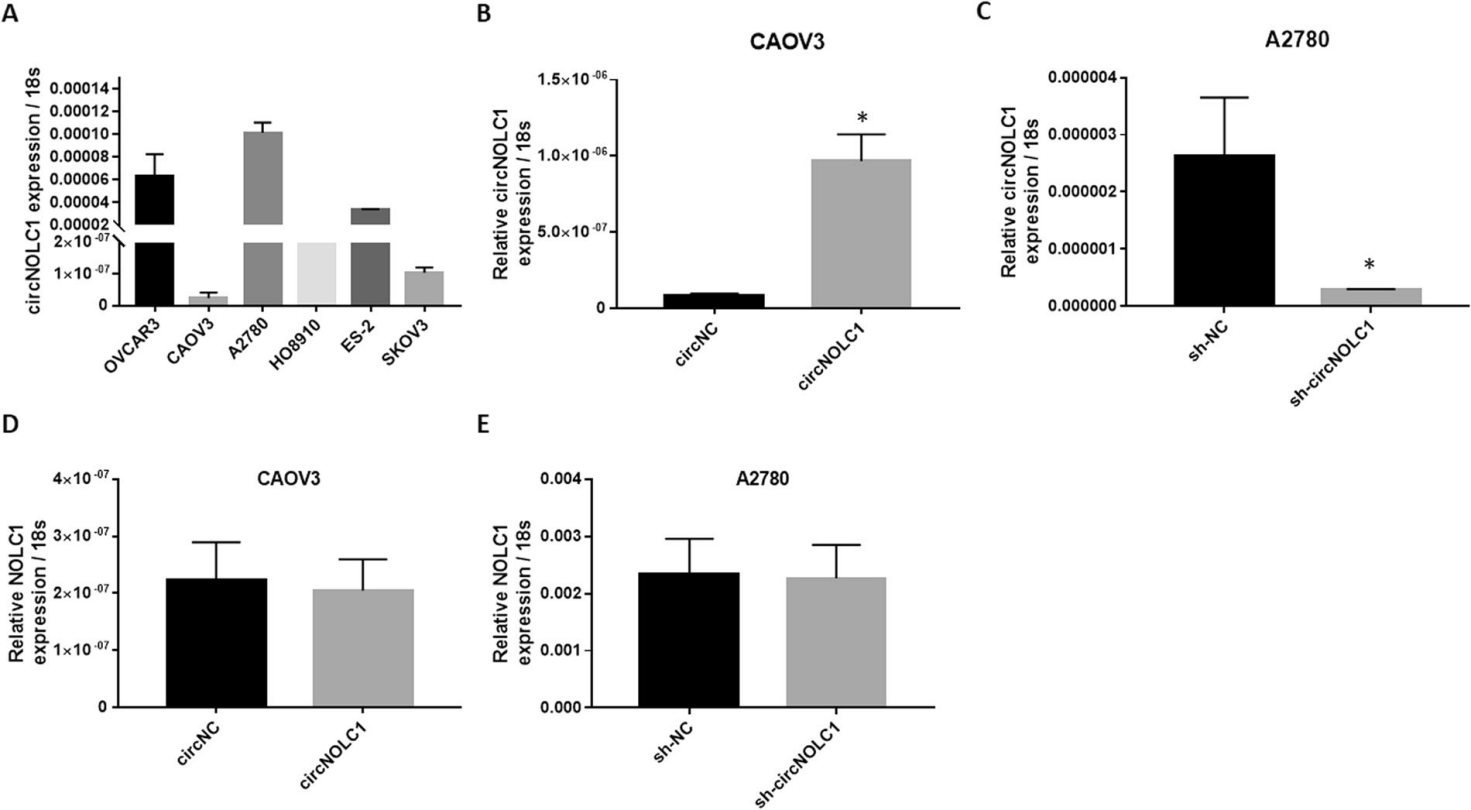
Circ-NOLC1 functions independently from the NOLC1 gene. **A** *Circ-NOLC1* expression in ovarian cancer cell lines was analyzed by qRT-PCR. Transcription efficiency was analyzed through qRT-PCR after *circ-NOLC1* overexpression plasmid transfection **B** or *Circ-NOLC1* shRNA transfection (**C**. The *NOLC1* mRNA expression level did not change neither after *circ-NOLC1* overexpression **D** nor downregulation (**E**). Results are representative of three separate experiments; data are expressed as the mean ± SD. Comparisons between two groups were analyzed using a two-sided Student’s *t* test. *p < 0.05.

We determined *circ-NOLC1* expression in six ovarian cancer cell lines using qRT-PCR. *Circ-NOLC1* showed the highest expression level in A2780 cells and the lowest in CAOV3 cells (Fig. 2A); therefore, A2780 cells were used for *circ-NOLC1* short hairpin RNA (shRNA) transfection, whereas CAOV3 cells were used for *circ-NOLC1* overexpression plasmid transfection. QRT-PCR was used to confirm the transfection efficiency (Fig. 2B, C, *p < 0.05). Besides, we found that the *NOLC1* mRNA expression level did not change after *circ-NOLC1* overexpression (Fig. 2D, *p* > 0.05) or downregulation (Fig. 2E, *p* > 0.05), thus we speculated that *circ-NOLC1* functions independently of the NOLC1 gene.

### The effect of circ-NOLC1 in vitro and in vivo

**Fig. 3:**
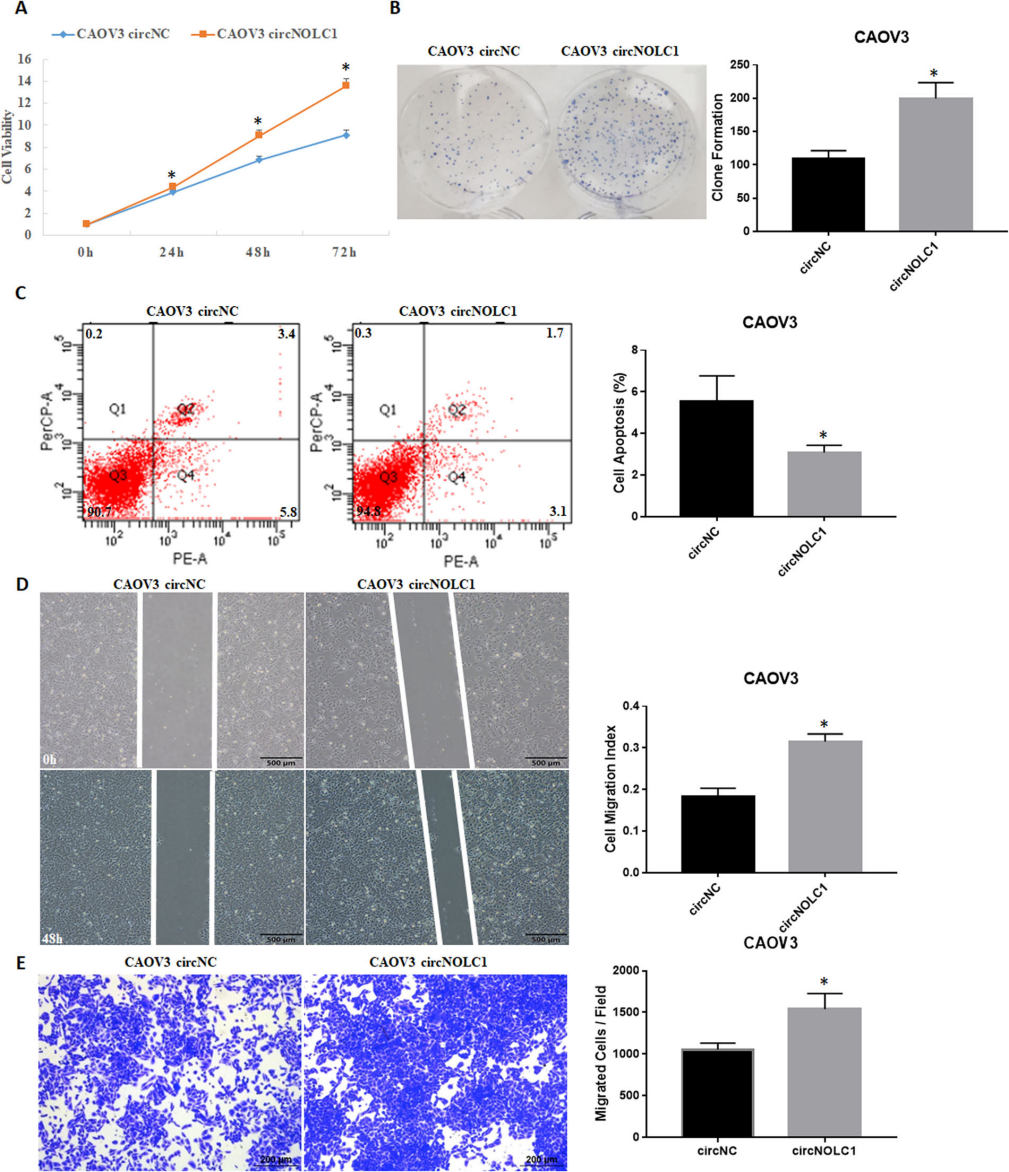
The effects of circ-NOLC1 overexpression in ovarian cancer cells. *Circ-NOLC1* overexpression induced cell proliferation **A** and clonal formation **B**; reduced cell apoptosis **C**; and induced cell migration **D** and invasion ability (**E**). The results are representative of three separate experiments; data are expressed as the mean ± SD. Comparisons between two groups were analyzed using a two-sided Student’s *t* test. *p < 0.05.

**Fig. 4:**
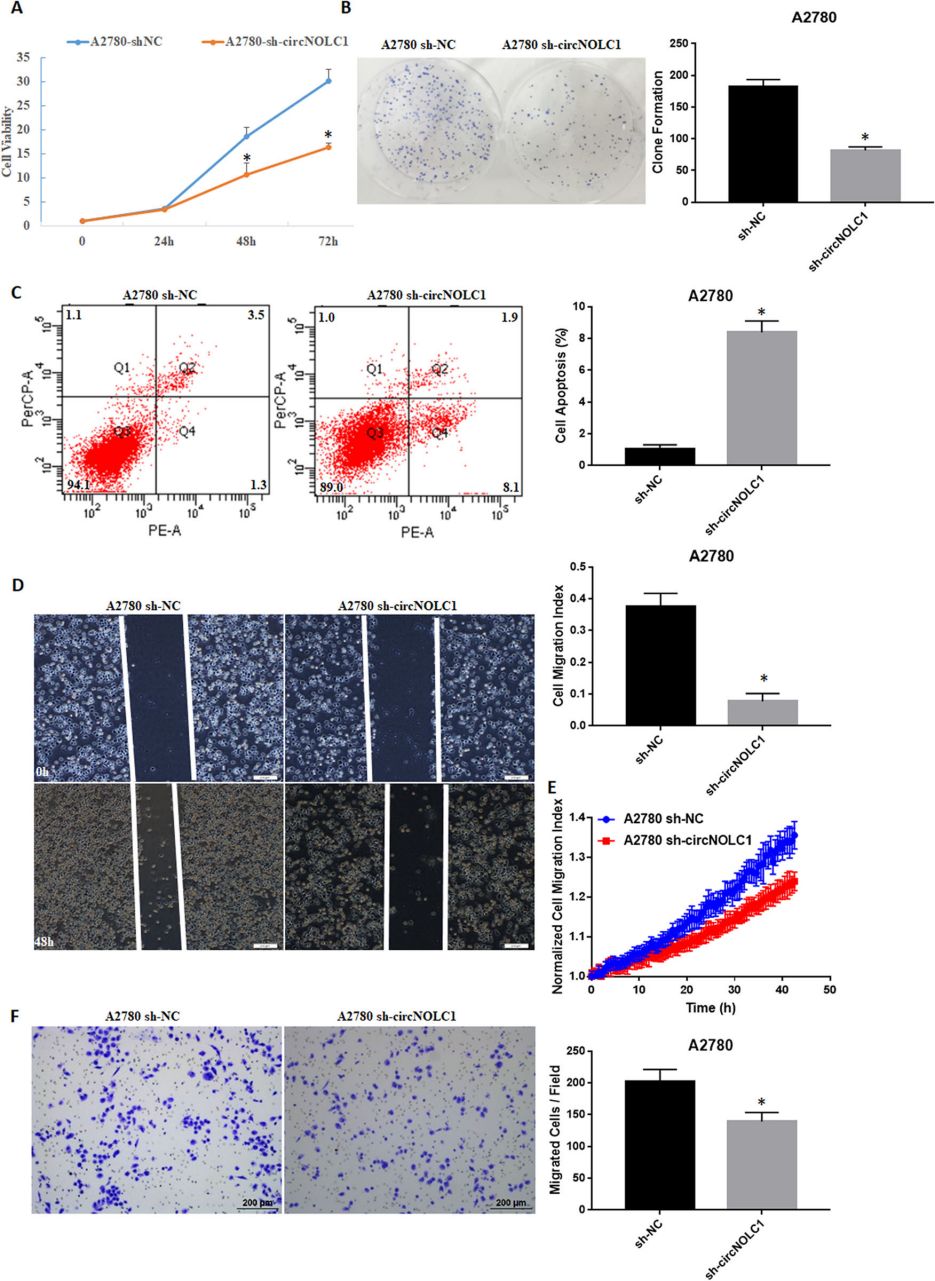
The effects of circ-NOLC1 downregulation in ovarian cancer cells. *Circ-NOLC1* downregulation reduced cell proliferation **A** and clonal formation **B**; induced cell apoptosis **C**; and reduced cell migration **D** & (**E**, Supplementary video 1: A2780 sh-NC, Supplementary video 2: A2780 sh-circ-NOLC1) and invasion (**F**). The results are representative of three separate experiments; data are expressed as the mean ± SD. Comparisons between two groups were analyzed using a two-sided Student’s *t* test. *p < 0.05.

**Fig. 5:**
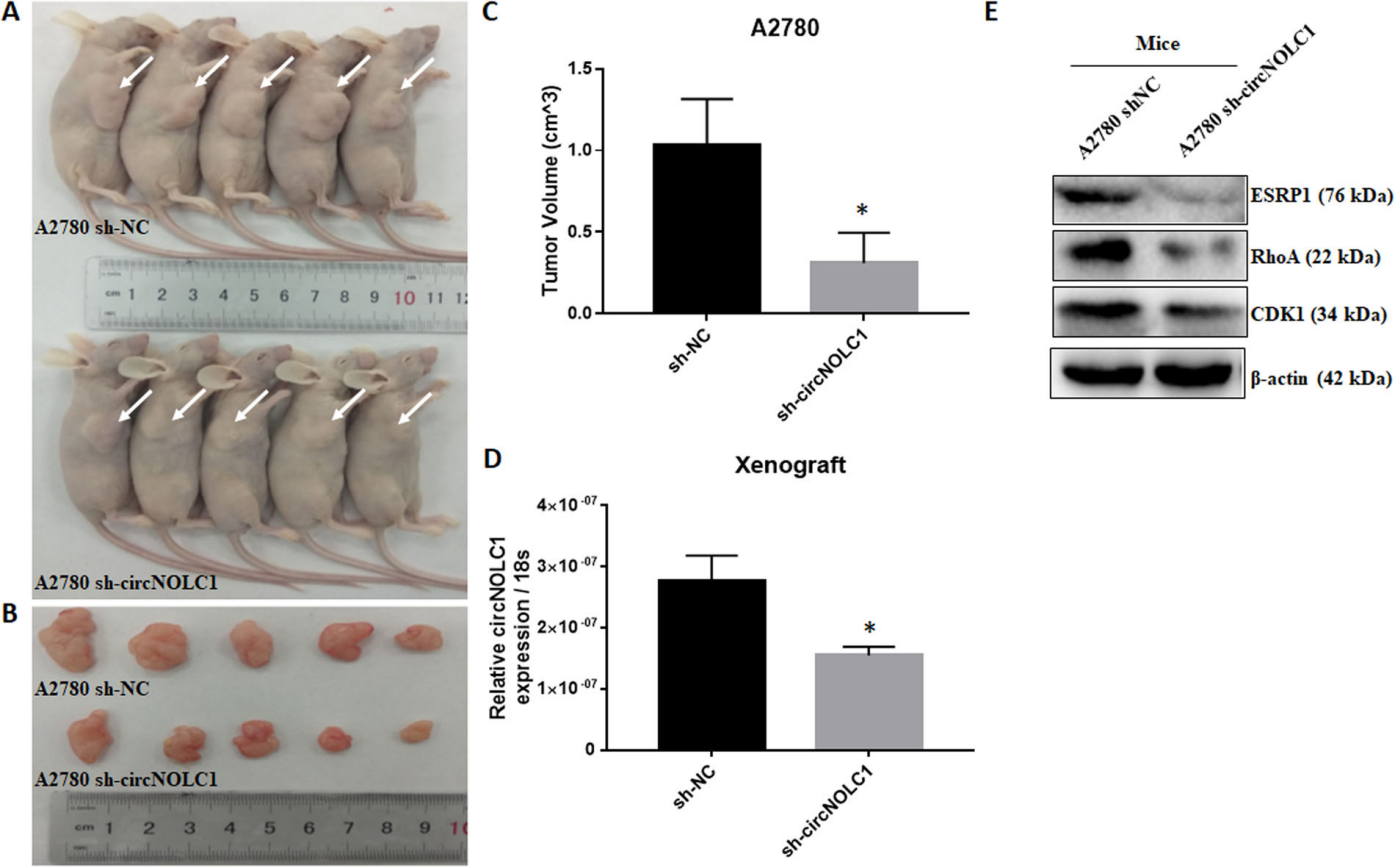
The effect of circ-NOLC1 downregulation in vivo. *Circ-NOLC1* downregulation reduced tumorigenicity in nude mice (**A** & **B**). The tumor volumes were smaller in the sh-circ-NOLC1 group than in sh-NC group (**C**), *Circ-NOLC1* expression (**D**) and ESRP1, RhoA, CDK1 protein level were all downregulated in sh-circ-NOLC1 group than in sh-NC group (**E**). Comparisons between two groups were analyzed using a two-sided Student’s *t* test. *p < 0.05.

Ovarian cancer cell proliferation, migration, and invasion were assessed through CCK8, plate clone, cell apoptosis, wound healing, and transwell assays. The results indicated that *circ-NOLC1* overexpression in CAOV3 cells significantly induced cell proliferation (Fig. 3A, *p* < 0.05) and clonal formation (Fig. 3B, *p* < 0.05); reduced cell apoptosis (Fig. 3C, *p* < 0.05); and induced cell migration (Fig. 3D, *p* < 0.05) and invasion (Fig. 3E, *p* < 0.05), which means that *circ-NOLC1* functions as an “oncogene” in ovarian cancer. Compared with the negative control, after *circ-NOLC1* downregulation using *circ-NOLC1* shRNA transfection in A2780 cells, cell proliferation (Fig. 4A, *p* < 0.05) and clonal formation (Fig. 4B, *p* < 0.05) were significantly inhibited, whereas cell apoptosis was induced (Fig. 4C, *p* < 0.05), and cell migration (Fig. 4D, E, *p* < 0.05, Supplementary videos 1 & 2) and invasion (Fig. 4F, *p* < 0.05) were decreased. Nude mice xenograft assays demonstrated that the tumorigenicity was inhibited in mice injected with *circ-NOLC1*-downregulated A2780 cells (Fig. 5D; *p* < 0.05) compared with that in the control group (Fig. 5A, B), and the tumor volumes were smaller in the *circ-NOLC1* downregulation group (Fig. 5C; *p* < 0.05).

### The correlation between circ-NOLC1 and ESRP1

**Fig. 6:**
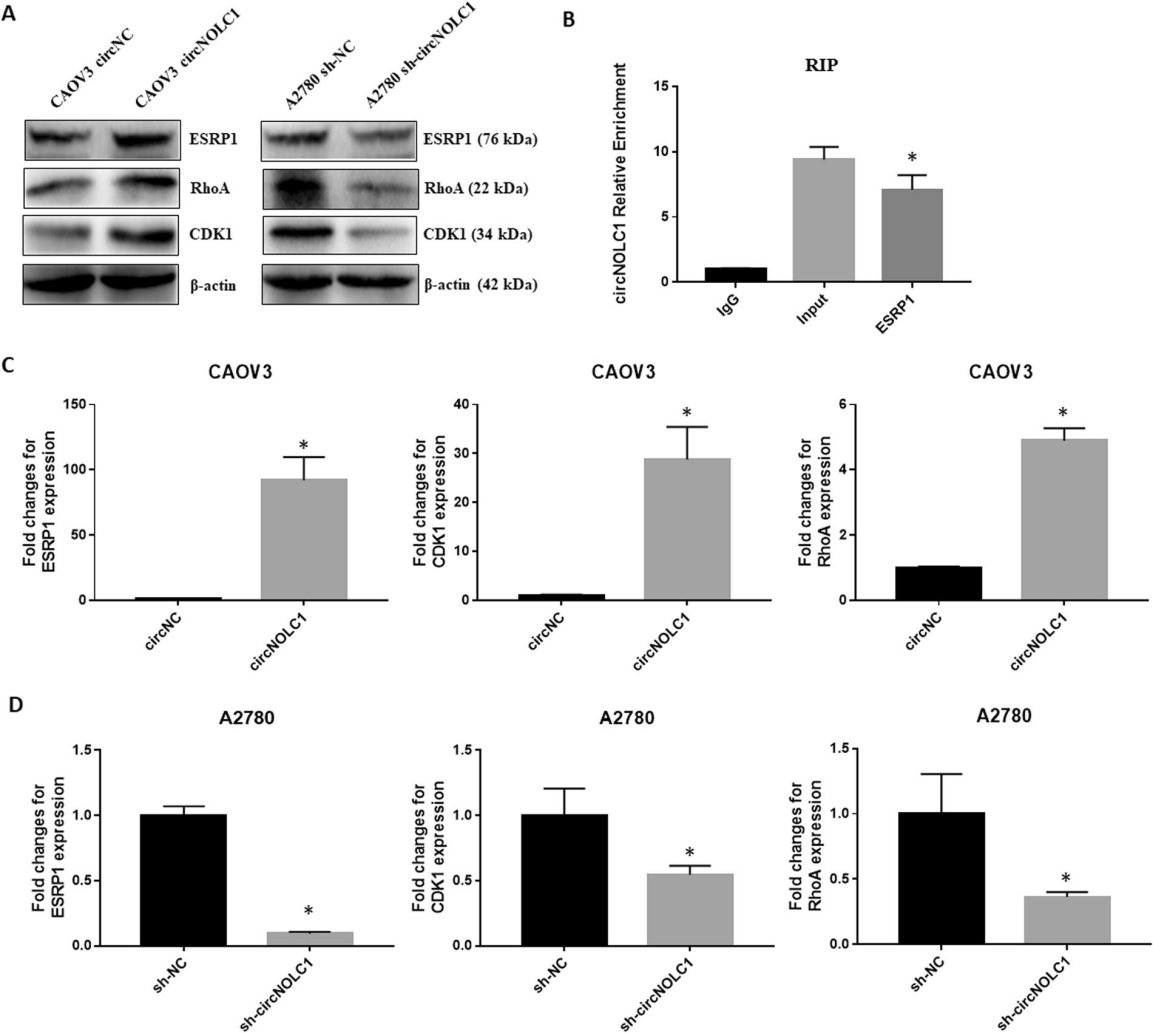
The correlation between circ-NOLC1 and ESRP1. *Circ-NOLC1* overexpression or downregulation induced or inhibited ESRP1, RhoA and CDK1 protein **A** or mRNA expression **C**–**D**, respectively. RIP assay confirmed the enrichment of *circ-NOLC1* (**B**). Comparisons between two groups were analyzed using a two-sided Student’s *t* test. *p < 0.05.

Bioinformatic prediction showed that *circ-NOLC1* has binding sites for miR-326-5p, miR-330, miR-370, and miR-9-5p (Circular RNA Interactome, https://circinteractome.irp.nia.nih.gov/), and these miRNAs also have binding sites for RhoA and CDK1 mRNA (mirDIP, http://ophid.utoronto.ca/mirDIP/index.jsp). Using western blotting, we confirmed that *circ-NOLC1* overexpression induced RhoA and CDK1 protein expression, while *circ-NOLC1* downregulation inhibited RhoA and CDK1 protein expression both in vivo and in vitro (Figs. 5E and 6A). However, the results of dual-luciferase reporter assay indicated that *circ-NOLC1* might not bind with miR-326-5p, miR-330, miR-370, and miR-9-5p (Supplementary Fig. 1). Bioinformatic prediction software (catRAPID, http://s.tartaglialab.com/page/catrapid_group) revealed that ESRP1 could bind with *circ-NOLC1* (Supplementary Fig. 2), which we confirmed experimentally using a RIP assay. RNA obtained from the RIP assay using an anti-ESRP1 antibody was subjected to qPCR analysis, which demonstrated the enrichment of *circ-NOLC1* (Fig. 6B). We also confirmed that *circ-NOLC1* overexpression could induce ESRP1 protein expression, whereas circ-NOLC1 downregulation inhibited ESRP1 protein expression. Besides, through qRT-PCR, we confirmed that *circ-NOLC1* overexpression induced ESRP1, RhoA, and CDK1 mRNA expression, whereas *circ-NOLC1* downregulation inhibited ESRP1, RhoA, and CDK1 mRNA expression (Fig. 6C, D).

### Silencing ESRP1, CDK1 or RhoA could reverse circ-NOLC1’s function as an oncogene

**Fig. 7:**
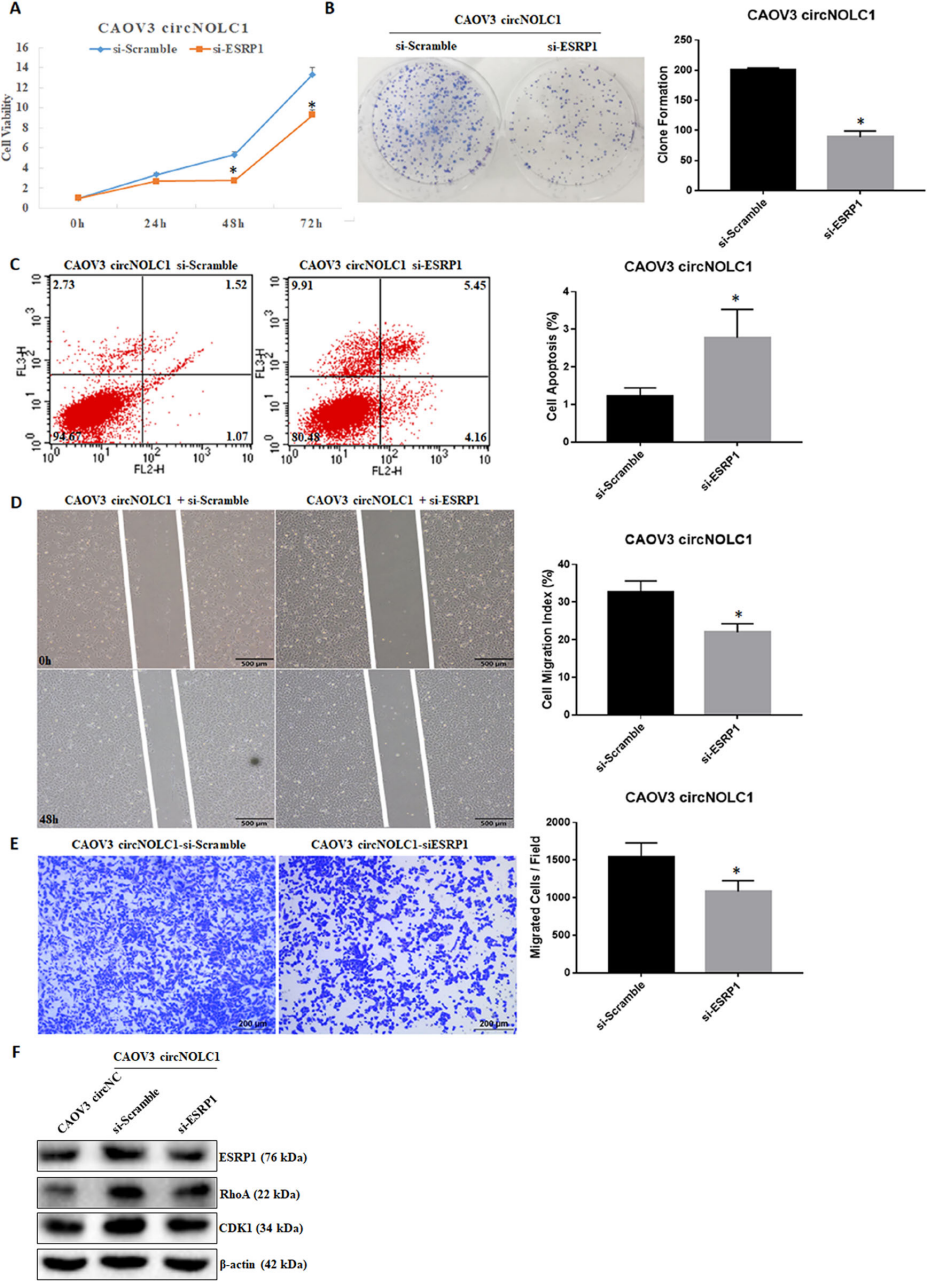
Silencing ESRP1 reversed circ-NOLC1’s function as an oncogene. Silencing ESRP1 inhibited cell viability **A** and clonal formation **B**, induced apoptosis **C**, and reduced cell migration **D** and invasion **E** in *circ-NOLC1*-overexpressing CAOV3 cells, and inhibited RhoA and CDK1 protein expression (**F**). Results are representative of three separate experiments; data are expressed as the mean ± SD. Comparisons between two groups were analyzed using a two-sided Student’s *t* test. *p < 0.05.

**Fig. 8:**
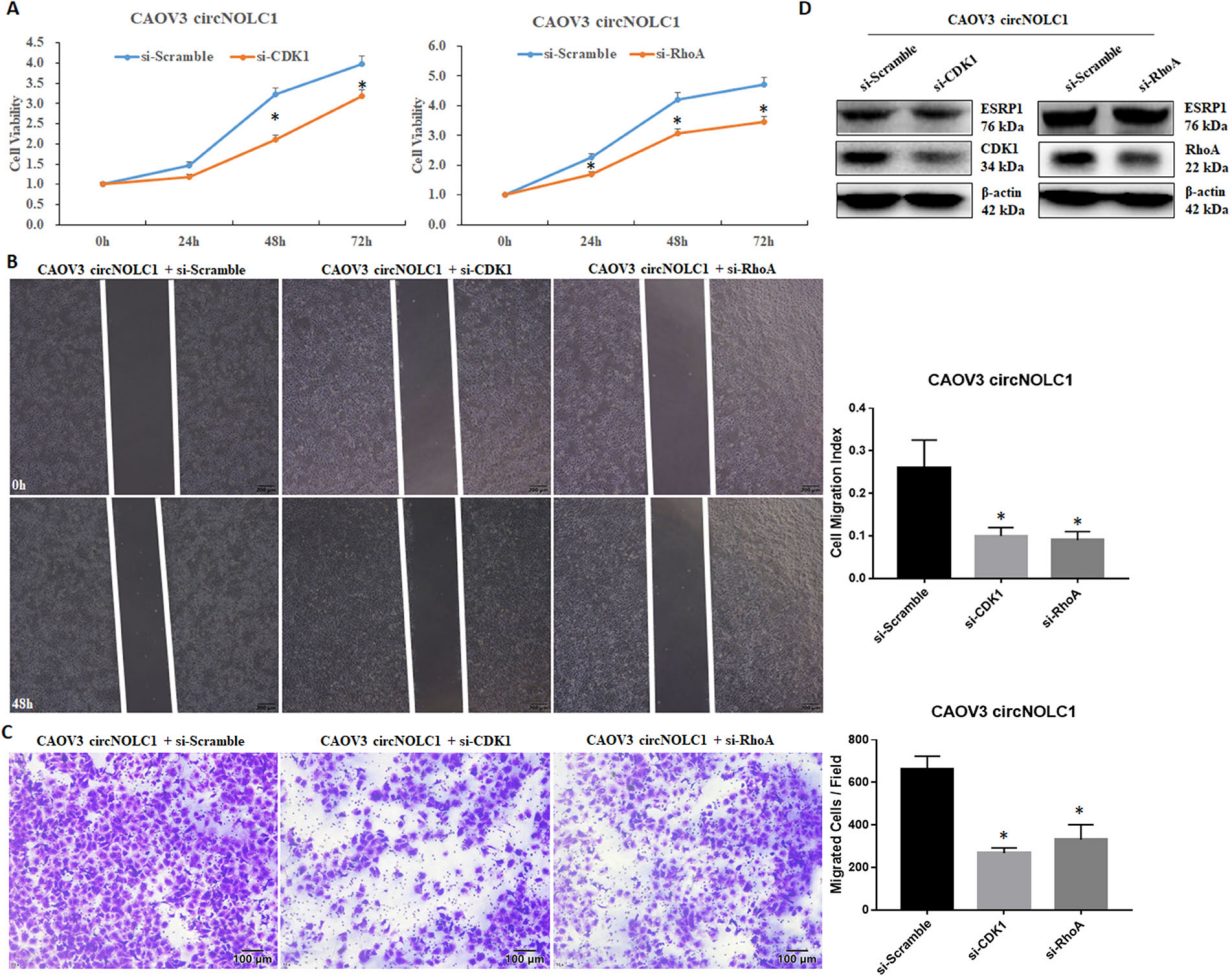
Silencing CDK1 or RhoA could also reverse circ-NOLC1’s function as an oncogene. Silencing *CDK1* or *RhoA* in *circ-NOLC1*-overexpressing CAOV3 cells significantly reduced cell viability **A**, cell migration **B**, and invasion **C** ability. *CDK1* or *RhoA* knockdown reduced CDK1 or RhoA protein expression, but not ESRP1 protein expression (**D**). Results are representative of three separate experiments; data are expressed as the mean ± SD. Comparisons between two groups were analyzed using a two-sided Student’s *t* test. *p < 0.05.

Silencing *ESRP1* in *circ-NOLC1*-overexpressing CAOV3 cells significantly reduced cell viability (Fig. 7A, *p* < 0.05) and clonal formation (Fig. 7B, *p* < 0.05), induced apoptosis (Fig. 7C, *p* < 0.05), and reduced cell migration (Fig. 7D, *p* < 0.05) and invasion (Fig. 7E, *p* < 0.05). Furthermore, knockdown of *ESRP1* reversed the ability of *circ-NOLC1* to upregulate RhoA and CDK1 protein expression in circ-NOLC1-overexpressing CAOV3 cells (Fig. 7F). These results showed that ESRP1 has a key role during the tumor-promoting process of *circ-NOLC1*. What’s more, we also found significantly reduced cell viability (Fig. 8A, *p* < 0.05), cell migration (Fig. 8B, *p* < 0.05) and invasion (Fig. 8C, *p* < 0.05) ability neither after *CDK1* or RhoA downregulation in *circ-NOLC1*-overexpressing CAOV3 cells, however, ESRP1 protein expression level did not change after neither after *CDK1* or *RhoA* knockdown (Fig. 8D). Thus, we hypothesized that *circ-NOLC1* might participate in ovarian cancer tumorigenesis and progression by binding the ESRP1, thus modulating RhoA and CDK1 expression.

## Discussion

The results of the present study have showed that *circ-NOLC1* expression was significantly higher in ovarian cancer tissues than in normal ovarian tissues. *Circ-NOLC1* expression in FIGO stage II–IV disease was higher than that in stage I, and in moderate and poor differentiation than in the well differentiation ovarian cancer tissues. Besides, the expression of *circ-NOLC1* was also higher in CA125 positive ovarian cancer patients. Thus, we hypothesized that circ-NOLC1 might participate in ovarian cancer tumorigenesis and progression.

Furthermore, in vitro, *circ-NOLC1* overexpression induced cell growth, migration, and invasion, whereas silencing of *circ-NOLC1* decreased cell growth, migration, and invasion. In addition, nude mice xenograft assays also demonstrated reduced tumorigenicity in mice injected with circ-NOLC1-downregulated A2780 cells. However, neither *circ-NOLC1* downregulation nor overexpression influence *NOLC1* mRNA expression. Thus, we hypothesized that *circ-NOLC1* functions independently of NOLC1, which prompted the question: how does *circ-NOLC1* function as an oncogene?

CircRNAs have attracted much attention recently, and studies have shown that circRNAs participate in cancer development in many ways. The most common function of circRNAs is as miRNA sponges, thereby releasing the inhibition of the miRNAs’ target mRNAs. Through bioinformatic analysis, we found that *circ-NOLC1* has binding sites for a series of miRNAs such as miR-326-5p, miR-330, miR-370, and miR-9-5p, and that these miRNA also have binding sites with RhoA and CDK1.

Ras homologous family member A (RhoA) is a small molecule G protein/GTPase (~22 kDa)^21^ that regulates the cytoskeleton and affects cell migration by activating ROCK and other effector proteins^22^. Studies have shown that this mode of regulation is involved in the invasion and metastasis of various tumors, such as gastric cancer^23^ and breast cancer^24^. Our previous research found that RhoA is overexpressed in ovarian epithelial cancer, and its expression is positively associated with FIGO stage and differentiation. RhoA not only affects ovarian cancer invasion and metastasis, but also affects the occurrence of ovarian cancer by regulating apoptosis-related genes^25^. Cyclin-dependent kinase 1 (CDK1 or CDC2), a member of the serine/threonine protein kinase family, is the checkpoint protein for the cell cycle from G2 to M phase and is involved in a variety of tumorigenic developments^26^. Our previous research also proved that CDK1 participates in ovarian cancer tumorigenesis and progression^27^. We first assessed RhoA and CDK1 expression levels after *circ-NOLC1* overexpression and downregulation. After *circ-NOLC1* overexpression or downregulation, RhoA and CDK1 protein levels were significantly upregulated or inhibited, respectively. Thus, we hypothesized that *circ-NOLC1* could promote ovarian cancer development by binding with miRNAs that inhibit the translation of CDK1 and RhoA. However, dual-luciferase reporter assays demonstrated that *circ-NOLC1* might not directly bind with miR-326-5p, miR-330, miR-370, or miR-9-5p, leaving open the question of how *circ-NOLC1* influences RhoA and CDK1 expression.

Studies have shown that despite functioning as miRNA sponges, circRNAs might also function in other ways, such as regulating transcription and acting as enzyme scaffolds. Recently, researchers showed that circRNAs could function by enabling circRNA-protein interactions^28^. For example, Abdelmohsen et al.^29^ reported that the extensive binding of Hu-Antigen R (HuR) to *circ-PABPN1* prevents its binding to poly(A) binding protein nuclear 1 (*PABPN1*) mRNA and reduces PABPN1 translation. In addition, our previous research reported that *circRhoC* functions not only as a miR-302e sponge to positively regulate VEGFA protein expression, but may also directly bind and modulate VEGFA expression to promote ovarian cancer development^30^. Recently, Sun et al.^31^ reported that circMYBL2 regulates the translation efficiency of oncogene FLT3 mRNA by recruiting RNA-binding protein PTBP1, thereby promoting the occurrence and development of FLT3-ITD mutant leukemia, which demonstrated for the first time that circRNA could play a positive regulatory role in the translation process in the form of an RNA-protein complex. Using bioinformatic analysis, we found that *circ-NOLC1* could also bind with proteins such as ESRP1. An extensive literature search revealed that ESRP1 was also overexpressed in ovarian cancer tissues; therefore, we suggested that *circ-NOLC1* could bind with ESRP1 and modulating CDK1 and RhoA expression. Through RIP assay we confirmed that *circ-NOLC1* binds with ESRP1. In support of this, we found that *circ-NOLC1* overexpression increased the ESRP1 protein level, whereas *circ-NOLC1* downregulation has the opposite effect. In addition, the tumor-promoting effect of *circ-NOLC1* was reduced after *ESRP1, CDK1, or RhoA* knockdown in *circ-NOLC1*-overexpressing cells, thus we hypothesized that *circ-NOLC1* might function as an oncogene through binding and modulating ESRP1, CDK1, and RhoA levels.

ESRP1 is an epithelial cell-specific RNA-binding protein that regulates alternative splicing of multiple genes. Studies indicate that ESRP1 has a critical role in tumor motility and invasiveness by facilitating cancer cell escape from primary tumors^32^. Studies also showed that ESRP1 promotes breast cancer metastasis by regulating *CD44* mRNA alternative splicing. Besides, high ESRP1 expression indicates a shorter overall survival^33^. Fagoonee et al.^34^ reported that the overexpression of ESRP1 promotes colorectal cancer development by stimulating cell proliferation. Jeong et al.^35^ reported that ESRP1 is overexpressed in ovarian cancer and participates in the epithelial-mesenchymal transition process. In addition, after inhibiting ESRP1 expression in *circ-NOLC1* overexpressing CAOV3 cells, RhoA and CDK1 protein levels were decreased, while CDK1 or RhoA downregulation has no effect on ESRP1 protein level, which confirmed our suggestion that *circ-NOLC1* functions as an oncogene by binding ESRP1 and modulating CDK1 and RhoA levels. *Circ-NOLC1* may bind to ESRP1 protein, and inhibit protein degradation by affecting its protein stabilization in the form of an RNA-protein complex, thereby promoting the expression of downstream CDK1 and RhoA.

## Conclusions

We demonstrated that *circ-NOLC1* promotes ovarian cancer tumorigenesis and development by binding to ESRP1 and modulating CDK1 and RhoA levels. This is the first study to suggest that *circ-NOLC1* functions in ovarian cancer and to propose its potential molecular mechanism. Thus, our findings provide a new perspective for the early diagnosis and targeted therapy of ovarian cancer.

## Materials and methods

### Ovarian cancer specimens

Normal ovaries, benign ovarian tumors, borderline tumors, and ovarian cancer tissues were collected from patients who accept gynecological surgery at the First Affiliated Hospital of China Medical University (Shenyang, China). After collection, samples were frozen in liquid nitrogen immediately. The tissue specimen was confirmed by two pathologists independently. The study was approved by the Ethics Committee of the China Medical University (no: 2018-132).

### Cell culture and transfection

Human ovarian cancer cell lines A2780, CAOV3, ES-2, HO8910, OVCAR3, and SKOV3 were purchased from Jennio Biotech (Guangzhou, China) or ATCC (Manassas, VA, USA). A2780 and ES-2 cells were cultured in Dulbecco’s modified Eagle’s medium (HyClone, Logan, UT, USA); SKOV3 cells were cultured in McCoys’ 5 A; whereas CAOV3, OVCAR3, and HO8910 cells were cultured in Roswell Park Memorial Institute-1640 (HyClone). All media were supplemented with 10% fetal bovine serum (FBS) and penicillin/streptomycin (100 U/mL). The cells were incubated with 5% CO_2_ at 37 °C. Lipofectamine 3000 was used for plasmid or small interfering RNA transfection according to the manufacturer’s instructions (Invitrogen, Carlsbad, CA, USA). *Circ-NOLC1* was upregulated using a *circ-NOLC1* overexpressing plasmid, and was knocked down using a *circ-NOLC1* shRNA expressing plasmid (the sequence is shown in the Supplemental file). Puromycin was used to select stable clonal cell lines. The sequence of si-ESRP1 was 5′-CUGAAGAAGUGGUGGCCUUdTdT-3′, and 3′-AAGGCCACCACUUCUUCAGdTdT-5′.

### CCK8 (Cell Counting Kit-8) assay

Cell viability was examined using the CCK8 assay. Cells were resuspended and seeded in 96-well plates at a density of 1500 cells per well. After the cells were plated, 10 μL of CCK8 solution (Bintech Co., Ltd., Shanghai, China) was added into each well at 0, 24, 48, and 72 h, and then incubated in 5% CO_2_ and 37 °C for 2 h. The absorbance at 450 nm was measured using a microplate spectrophotometer (BioTek Instruments, Winooski, VT, USA).

### Wound-healing assay

Cell migration was detected using a wound-healing assay. The cells were resuspended and seeded in six-well plates at a density of 5 × 10^5^ cells per well. When the cells reached 80% confluence, each well was scored vertically using a 200-μL pipette tip. The excess suspended cells were then rinsed off using phosphate-buffered saline (PBS) three times. Two milliliters of the serum-free medium was then added to each well and the cells were incubated at 5% CO_2_ and 37 °C. The wounded cells were photographed under an optical microscope at 0, 24, and 48 h, and the wound area was measured using Image J software (National Institutes of Health, Bethesda, MD, USA). The wound-healing rate was calculated as follows: (original wound area−wound area at each time point)/original wound area × 100%.

### Cell migration assay by using cytation 5 cell imaging multi-mode reader

Cells were plated (40,000 cells/well) in white, 96-well clear-bottom plates (Corning) with 100 μL final volume of complete media. When the cells were plated, AutoScratch^TM^ (BioTek) was used to form scratch, the excess suspended cells were then rinsed off using PBS three times, after which, 100 μL serum-free medium was then added to each well and the 96-well plate was incubated and imaged (every 30 min) at 5% CO_2_ and 37 °C using a Cytation 5 Cell Imaging Multi-Mode Reader (BioTek Instruments, Winooski, VT, USA).

### Invasion assay

The cell invasion ability was measured using a Transwell chamber. Matrigel matrix (BD Biosciences, San Jose, CA, USA) was diluted 1:10 with serum-free medium. The prepared diluted Matrigel was then uniformly added to the upper chamber, and then the Transwell chamber was placed in an incubator for 3–4 h for coagulation. Then, 200 μL of serum-free medium containing 50,000 resuspended cells was added to the upper chamber, 600 μL of complete cell culture medium was added as a chemoattractant to the lower chamber, and after incubation for 48 h, the chambers were washed with PBS three times, fixed with 5% paraformaldehyde for 15 min, and then stained with a crystal violet solution for 15 min. Finally, the cells were counted under a microscope (Olympus, Tokyo, Japan) to evaluate cell invasion.

### Plate clone formation assay

Cells (200 cells per well) were seeded into a six-well plate, and then incubated in 5% CO_2_ and 37 °C for 2 weeks (complete medium) until macroscopic cell clones appeared. The cells were carefully washed using PBS, fixed using 4% paraformaldehyde for 15 min, stained with Giemsa solution for 15 min, washed with tap water, and air-dried. The colony formation rate was calculated using the following formula: (number of clones/number of cells inoculated) × 100%.

### Apoptosis assays

Cells were collected and resuspended with 100 μL 1× buffer containing phycoerythrin-labeled annexin V and 7-aminoactinomycin D (5 μL each, BD) in the dark for 15 min. Then, 400 μL of buffer was added and the rate of cell apoptosis was determined using flow cytometry within one hour.

### QRT-PCR

Total RNA of tissue and cell samples was extracted using the TRIzol reagent (Takara, Shiga, Japan). Chloroform (200 μL) was added into 1 mL of TRIzol, and after centrifugation, the upper aqueous phase was aspirated into a new tube and an equal volume of isopropanol was added to precipitate the RNA. After centrifugation, the supernatant was discarded and 75% ethanol was used to wash the RNA pellet. The precipitate was dried and dissolved in diethylpyrocarbonate water. The OD value at 260 nm was measured using an ultraviolet spectrophotometer (Unico, Shanghai, China) to calculate the concentration of RNA. Then, RNA was reverse-transcribed to cDNA according to the manufacturer’s instructions (Takara). Next, real-time quantitative PCR amplification of cDNA was carried out using a SYBR PrimeX EX-TAQ Patent II Kit (Takara, Shiga, Japan). Finally, the cycle threshold (Ct) of the target gene and 18 S rRNA (18 s) were compared using the 2-ΔΔCt method (GenePharma) to determine relative expression levels of the target gene.

### Western blotting

Cells were lysed with radioimmunoprecipitation assay buffer containing protease inhibitors. After quantification, 40 μg of denatured protein was separated using 10% sodium dodecyl sulfate-polyacrylamide gel electrophoresis, followed by electro-transfer onto a polyvinylidene difluoride membrane that had already been pre-activated in methanol. The membrane was then washed with tris-buffered saline and Tween 20 (TBST) for 1–2 min, blocked with 3% bovine serum albumin at room temperature for 1–2 h. Thereafter, the membranes were incubated with primary antibodies against ESRP1 (Cat No.: 21045-1-AP), ras homolog family member A (RhoA, Cat No.: 10749-1-AP), CDK1 (Cat No.: 19532-1-AP) (1:1000, Proteintech Group, Chicago, IL, USA) and β-actin (1:3000, Cat No.: 20536-1-AP, Proteintech Group) overnight at 4 °C. On the 2nd day, the membrane was washed with TBST three times, and then incubated with anti-rabbit secondary antibodies at room temperature for 2 h. After washing with TBST again three times, the immunoreactive proteins were visualized using the ECL system (Santa Cruz Biotechnology, Santa Cruz, CA, USA).

### RNA-binding protein immunoprecipitation (RIP) assay

Bioinformatic prediction (catRAPID, http://s.tartaglialab.com/page/catrapid_group) revealed that ESRP1 protein could bind with *circ-NOLC1*. The Magna RIP RNA-Binding Protein Immunoprecipitation Kit (Millipore, Bedford, MA, USA) was used for the RIP assay. In brief, A2780 cells were lysed in RIP lysis buffer and the cell extract was incubated with RIP buffer containing magnetic beads conjugated to human anti-ESRP1 antibodies or normal rabbit IgG as a negative control. Thereafter, to digest the proteins, the samples were incubated with proteinase K. The immunoprecipitated RNA was isolated and determined using qRT-PCR analysis. Control amplification was carried out on input RNA before immunoprecipitation, which was set as the positive control.

### Subcutaneous tumor dissemination assay

BALB/c nude mice (Vital River Laboratories, Beijing, China) were raised in a specific pathogen-free environment. In brief, after serpentine grouping by weight, 1 × 10^7^ circ-NOLC1-downregulated A2780 cells or control A2780 cells in 150 µL FBS-free media were injected into 5-week-old female mice subcutaneously to establish the subcutaneous dissemination model. At appropriate times after injection (Tumor size <2 cm), the mice were euthanized and the tumor nodes were resected and measured. All animal experiments were approved by China Medical University Animal Care and Use Committee and were carried out following the Guide for the Care and Use of Laboratory Animals (published by the National Institute of Health). No blinding was done.

### Dual-luciferase reporter assay

The wild-type or mutated PSI-check2 Dual-luciferase vectors containing miR-326-5p, miR-330, miR-370, and miR-9-5p binding sites on *circ-NOLC1* (Hanbio Biotechnology, Shanghai, China) were co-transfected with miR-326-5p, miR-330, miR-370, and miR-9-5p mimics or scrambled controls into the HEK-293T cells. Cell extracts were prepared to measure the luciferase activity using the Dual-Luciferase Reporter Assay System (Promega, Madison, WI, USA). The relative luciferase signal was determined by the normalization of firefly luciferase activity to that of Renilla luciferase.

### Statistical analysis

Statistical analyses were performed using SPSS 20.0 software (IBM Corp., Armonk, NY, USA). Experiments were repeated three times, and the mean ± SD was used in each group of data to reflect the overall parameters. Comparisons between two groups were analyzed using a two-sided Student’s *t* test. Correlation between circ-NOLC1 expression and clinicopathological characteristics of the patients with ovarian cancer were examined using the *χ*^2^ test. *P* < 0.05 was considered statistically significant.

## Supplementary information

Supplementary Figure Legends

Supplementary Materials

Supplementary Figure 1

Supplementary Figure 2

Supplementary video 1

Supplementary video 2
