# Supplementary material for: Circ-NOLC1 promotes epithelial ovarian cancer tumorigenesis and progression by binding ESRP1 and modulating CDK1 and RhoA expression
Source: Cell Death Discov. 2021 Jan 22;7:22. doi: 10.1038/s41420-020-00381-0 (PMC7822960; doi:10.1038/s41420-020-00381-0)
Supplement: Supplementary file 1 — Supplementary Figure Legends [file 41420_2020_381_MOESM1_ESM.docx]

**Supplementary Figure legends**

**Supplementary Figure 1: *circ-NOLC1 might not directly bind with miR-326-5p, miR-330, miR-370, or miR-9-5p***

Dual luciferase reporter assay indicated that *circ-NOLC1* might not bind with *miR-9-5p* (A)*, miR-326-5p* (B)*, miR-330* (C)*, or miR-370* (D)*.* Results are representative of three separate experiments; data are expressed as the mean ± SD. Comparisons between two groups were analyzed using a two-sided Student’s t test.

**Supplementary Figure 2: *The potential binding proteins for circ-NOLC1***

Bioinformatic prediction (catRAPID) revealed the potential binding proteins for *circ-NOLC1,* which include ESRP1.
