# Supplementary material for: Circ-NOLC1 promotes epithelial ovarian cancer tumorigenesis and progression by binding ESRP1 and modulating CDK1 and RhoA expression
Source: Cell Death Discov. 2021 Jan 22;7:22. doi: 10.1038/s41420-020-00381-0 (PMC7822960; doi:10.1038/s41420-020-00381-0)
Supplement: Supplementary file 2 — Supplementary Materials [file 41420_2020_381_MOESM2_ESM.docx]

Sequences for Plasmids

**hsa_circRNA-NOLC1 (hsa_circRNA_100674)**

CCCGAATGGGATTAATAGCAGAGCTCGTTTAGTGAACCGTCAGATCGCCTGGAGACGCCATCCACGCTGTTTTGACCTCCATAGAAGACACCGACTCTACTAGAGGATCTATTTCCGGTGAATTCAAAGTGCTGAGATTACAGGCGTGAGCCACCACCCCCGGCCCACTTTTTGTAAAGGTACGTACTAATGACTTTTTTTTTATACTTCAGACACAGCAGGATGCCAATGCCTCTTCCCTCTTAGACATCTATAGCTTCTGGCTCAAGTCTGCCAAGGTCCCAGAGCGAAAGTTACAGGCAAATGGACCAGTGGCTAAGAAAGCTAAGAAGAAGGCCTCATCCAGTGACAGTGAGGACAGCAGCGAGGAGGAGGAGGAAGTTCAAGGGCCTCCAGCAAAGAAGGCTGCTGTACCTGCCAAGCGAGTCGGTCTGCCTCCTGGGAAGGCTGCAGCCAAAGCATCAGAGAGTAGCAGCAGTGAAGAGTCCAGTGATGATGATGATGAGGAGGACCAAAAGAAACAGCCTGTCCAGAAGGGAGTTAAGCCCCAAGCCAAGGCAGCCAAAGCTCCTCCTAAGAAGGCCAAGAGCTCTGATTCTGATTCTGACTCAAGCTCCGAGGATGAGCCACCAAAGAACCAGAAGCCAAAGATAACACCTGTGACAGTTAAAGCTCAGACTAAAGCCCCTCCCAAACCAGGTAAGAAGCAAGGAAAAGAATTAGGCTCGGCACGGTAGCTCACACCTGTAATCCCAGCAGGATCCATCGATACTAGTAAGGATCTGCGATCGCTCCGGTGCCCGTCAGTGGGCAGAGCGCACATCGCCCACAGTCCCCGAGAAGTTGGGGGGAGGGGTCGGCAATTGAACGGGTGCCTAGAGAAGGTGGCGCGGGGTAAACTGGGAAAGTGATGTCGTGTACTGGCTCCGCCTTTTTCCCGAGGGTGGGGGAGAACCGTATATAAGTGCAGTAGTCGCCGTGAACGTTCTTTTTCGCAACG

**hsa_circRNA-NOLC1 shRNA**

CAATGAATTTCGATTTCTTGGCTTTATATATCTTGTGGAAAGGACGAGGATCCGCCCCTCCCAAACCAGACACAGCAGTTCAAGAGACTGCTGTGTCTGGTTTGGGAGGGGCTTTTTTCAATTCTAGTTATTAATAGTAATCAATTACGGGGTCATTAGTTCATAGCCCATATATGGAGTTCCGCGTTACATAACTTACGGTAAATGGCCCGCCTGGCTGACCGCCCAACGACCCCCGCCCATTGACGTCAATAATGACGTATGTTCCCATAGTAACGCCAATAGGGACTTTCCATTGACGTCAATGGGTGGAGTATTTACGGTAAACTGCCCACTTGGCAGTACATCAAGTGTATCATATGCCAAGTACGCCCCCTATTGACGTCAATGACGGTAAATG
