# Supplementary figures and images for: Circ-NOLC1 promotes epithelial ovarian cancer tumorigenesis and progression by binding ESRP1 and modulating CDK1 and RhoA expression
Source: Cell Death Discov. 2021 Jan 22;7:22. doi: 10.1038/s41420-020-00381-0 (PMC7822960; doi:10.1038/s41420-020-00381-0)

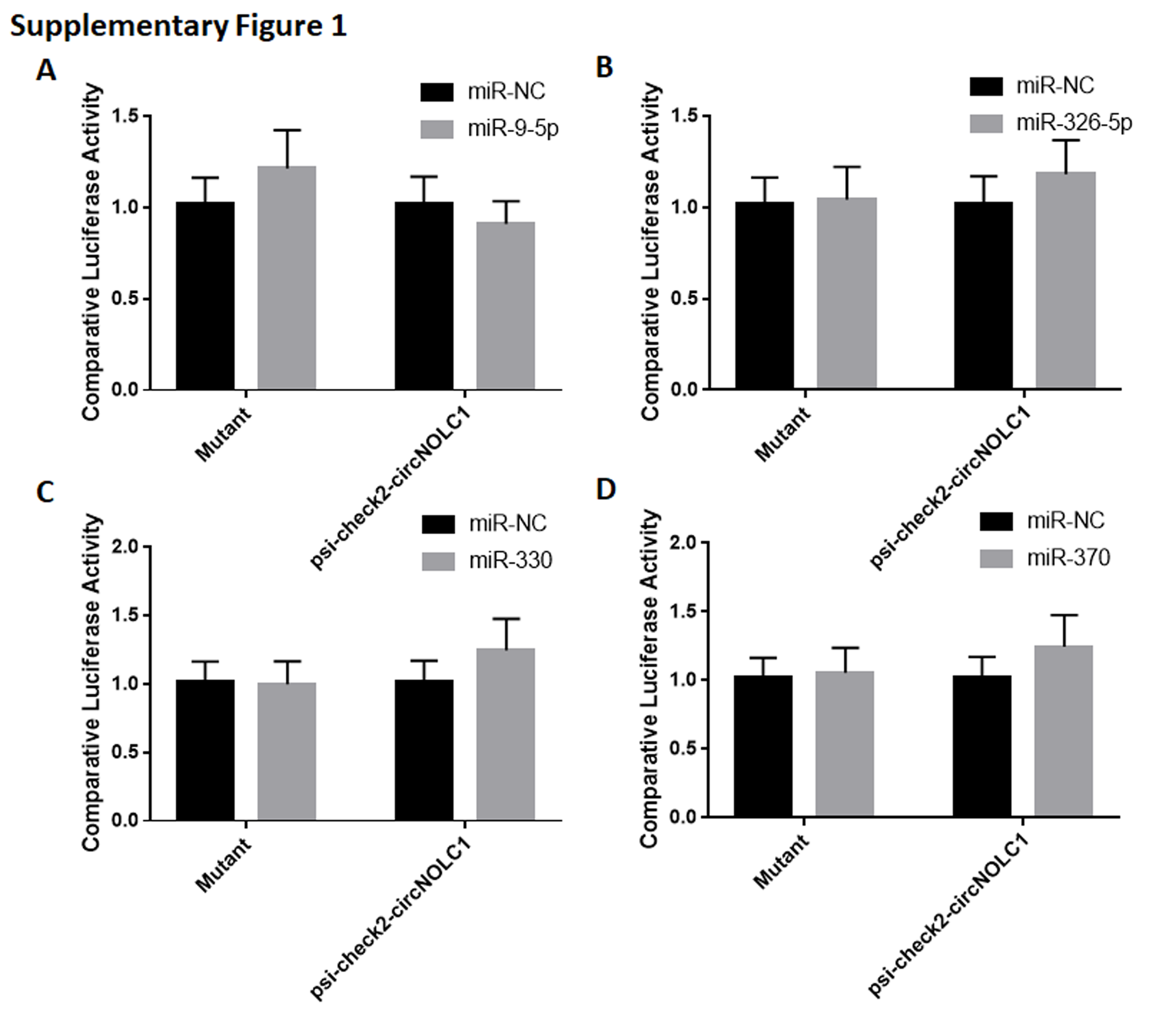

Supplement: Supplementary file 3 — Supplementary Figure 1 [file 41420_2020_381_MOESM3_ESM.tif]

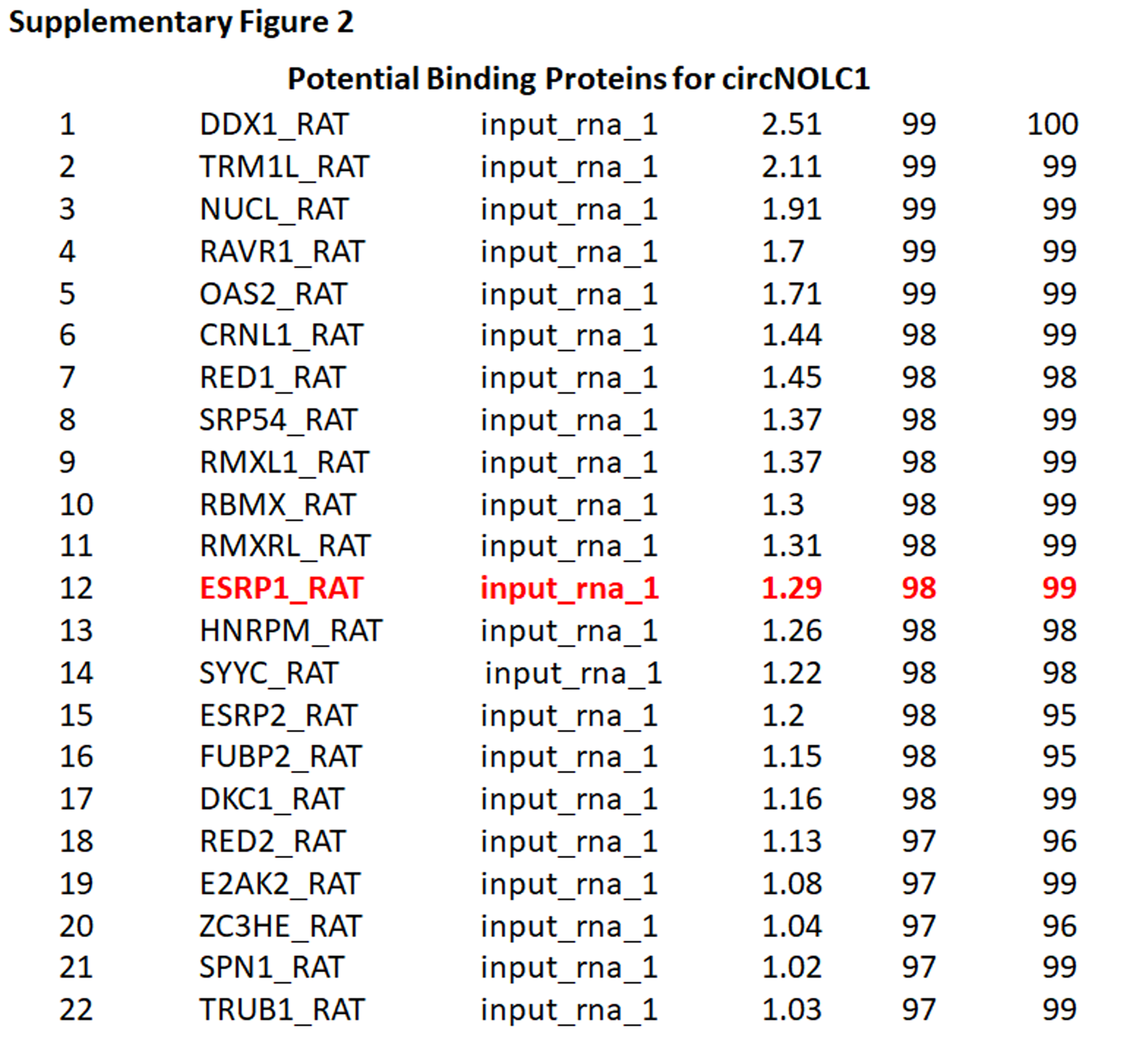

Supplement: Supplementary file 4 — Supplementary Figure 2 [file 41420_2020_381_MOESM4_ESM.tif]
